# Open and Lys–His Hexacoordinated Closed Structures of a Globin with Swapped Proximal and Distal Sites

**DOI:** 10.1038/srep11407

**Published:** 2015-06-22

**Authors:** Aik-Hong Teh, Jennifer A. Saito, Nazalan Najimudin, Maqsudul Alam

**Affiliations:** 1 Centre for Chemical Biology, Universiti Sains Malaysia, 10 Persiaran Bukit Jambul, 11900 Bayan Lepas, Penang, Malaysia; 2 Department of Microbiology, University of Hawaii, 2538 McCarthy Mall, Honolulu, HI 96822, United States; 3 School of Biological Sciences, Universiti Sains Malaysia, 11800 Penang, Malaysia; 4 Advanced Studies in Genomics, Proteomics and Bioinformatics, University of Hawaii, 2565 McCarthy Mall, Honolulu, HI 96822, United States

## Abstract

Globins are haem-binding proteins with a conserved fold made up of *α*-helices and can possess diverse properties. A putative globin-coupled sensor from *Methylacidiphilum infernorum*, HGbRL, contains an N-terminal globin domain whose open and closed structures reveal an untypical dimeric architecture. Helices E and F fuse into an elongated helix, resulting in a novel site-swapped globin fold made up of helices A–E, hence the distal site, from one subunit and helices F–H, the proximal site, from another. The open structure possesses a large cavity binding an imidazole molecule, while the closed structure forms a unique Lys–His hexacoordinated species, with the first turn of helix E unravelling to allow Lys52(E10) to bind to the haem. Ligand binding induces reorganization of loop CE, which is stabilized in the closed form, and helix E, triggering a large conformational movement in the open form. These provide a mechanical insight into how a signal may be relayed between the globin domain and the C-terminal domain of HGbRL, a Roadblock/LC7 domain. Comparison with HGbI, a closely related globin, further underlines the high degree of structural versatility that the globin fold is capable of, enabling it to perform a diversity of functions.

The bacterium *Methylacidiphilum infernorum*, isolated from the Hell’s Gate (Tikitere) geothermal area in New Zealand, is an aerobic methanotrophic bacterium growing optimally at 60 °C and pH 2.0^1^. Its genome encodes five globins — one double-domain HGbRL and four single-domain HGbI–IV^2^ — and thus far only the single-domain HGbI^3^ and HGbIV^4^ have been studied. HGbI possesses a very high affinity for O_2_ and an unusually low autoxidation rate^3^. Its structure consists of a typical but compact globin fold, with the highly conserved Tyr29(B10) and Gln50(E7) in the distal site flexible enough to bind a molecule of either O_2_ or acetate^3^. HGbIV, on the other hand, is a truncated haemoglobin with two unique extensions at both termini — a Pre-A loop at the N terminus and a helix I at the C terminus^4^. Its distal site, intriguingly, is comparatively large and polar, containing a specifically conserved His70(B9)–His71(B10) motif that in a crystal structure was well positioned to bind a phosphate ion^4^.

The globin fold, having one of the most versatile architecture, can bind a variety of different ligands in the distal site. Besides small ligands such as O_2_, CO and NO, ligands as large as econazole or even a phospholipid have been reported in the structures of yeast flavohaemoglobin^5^ (Yhb) and *Ralstonia eutropha* flavohaemoglobin (FHP)^6^,^7^,^8^. In some globins, their His(E7) bind directly to the haem in the absence of an exogenous ligand, forming a bis-His hexacoordinated species first observed in the structure of a nonsymbiotic plant haemoglobin^9^. Neuroglobin, meanwhile, employs a novel haem-sliding mechanism for its bis-His hexacoordination^10^. In the unligated structure of *Escherichia coli* flavohaemoglobin (HMP), interestingly, the sixth coordination site is physically occupied by Leu57(E11)^11^.

Globins have also been found fusing to the N termini of a variety of proteins, forming multi-domain proteins which are widely distributed in prokaryotes and eukaryotes alike. In flavohaemoglobins, which function in cellular responses to nitrosative stress, the globin domain is fused to a ferredoxin reductase-like module consisting of a FAD- and an NADH-binding domain^12^. In HemAT, a globin-coupled sensor (GCS) involved in *Bacillus subtilis* aerotaxis, the C-terminal domain is a methyl-accepting chemotaxis protein^13^. RsbR from *B. subtilis*, on the other hand, comprises a C-terminal STAS domain and forms a gigantic stressosome with RsbS and RsbT, which plays a role in the bacterium’s general stress response^14^. The N-terminal globin domain of RsbR, however, not only lacks helices C and D but also has the strictly conserved haem-binding His(F8) in the proximal site replaced by Ala74 — it no longer binds any haem^15^. While *B. subtilis* RsbR represents an extreme example of a globin that has evolved to lose its haem, the RsbR homologues from *Saprospira grandis* still retain their haem and are able to bind O_2_^16^.

HGbRL from *M. infernorum* is a putative globin-coupled sensor of 274 residues, consisting of an N-terminal globin domain and a C-terminal Roadblock/LC7 domain. It is unique to the genus *Methylacidiphilum*, and presently is found only in two other closely related bacteria, *M. kamchatkense*^17^ and *M. fumariolicum*^18^. The globin domain, *N*-HGbRL, is 38% identical to HGbI, with both belonging to the same group of bacterial globins that lack helix D and conserve both Tyr(B10) and Gln(E7) (Fig. 1). The structures of several bacterial globins from this group, such as HMP^11^, FHP^6^,^7^,^8^ and *Vitreoscilla stercoraria* haemoglobin (*Vs*Hb)^19^, have displayed a high degree of versatility in their distal sites involving mainly loop CE and helix E. HMP, for instance, moves its helix E inwards in the closed form to occupy the distal site^11^, while FHP moves its outwards in the open form, expanding the distal site to bind a ligand as large as a phospholipid^7^. Here we further demonstrate the architectural plasticity of the globin fold by reporting the structure of an unprecedented site-swapped dimeric globin, *N*-HGbRL, which has been solved in both the open and closed forms with several novel features.

## Results and Discussion

### Hexacoordination of *N*-HGbRL

The purifieddimeric *N*-HGbRL was in the ferric state, whose spectrum revealed a predominantly hexacoordinated form with a Soret band at 412 nm and a visible peak at 534 nm (Fig. 2). In the presence of an excess of sodium dithionite that also removed dissolved oxygen, *N*-HGbRL was reduced but in the hexacoordinated form too, with the Soret band shifting to 424 nm and two distinct peaks emerging at 529 and 557 nm (Fig. 2). Both HGbI^3^ and HGbIV^4^ have been reported to form a hexacoordinated species as well. The internal sixth ligand, however, did not seem to form a strong interaction with the haem at least in the ferrous state, as under the experimental conditions the deoxy *N*-HGbRL slowly turned oxygenated within 20 minutes, with the Soret band and the two peaks shifting to 413, 544 and 576 nm (Fig. 2).

### Site-swapped *N*-HGbRL with fused helices E and F

*N*-HGbRL consists of seven *α*-helices, helices A–H, lacks helix D and forms a dimer. It displays a typical bacterial globin fold except for a very untypical feature — its helices E and F unexpectedly fuse into an elongated helix (Fig. 3a). Loop EF is straightened into part of the *α*-helical conformation, and the elongated helices from both subunits form an antiparallel pair. Instead of being sandwiched between helices E and F from a single globin subunit, a haem group in *N*-HGbRL is sandwiched by two subunits. A single *N*-HGbRL globin fold, denoted subunit A*, is therefore made up of helices A–E from one subunit and helices F–H from the other. In other words, both the proximal and distal sites of a single globin fold are constituted mainly by two separate subunits, representing the first ever site-swapping example in the globin family. The two C-terminal Roadblock/LC7 domains, meanwhile, will be positioned at the opposite ends of the dimer’s helices H, but may still lie in the same side to interact with one another.

The structure of *N*-HGbRL was first solved in the open form in space group *C*2, with two subunits in the asymmetric unit. In the distal site, an imidazole molecule from the Ni^2+^-affinity chromatography binds to the haem and the highly conserved Tyr28(B10), which also coordinates a water molecule, W1 (Fig. 3b). Several imidazole-bound globin structures have been reported too, including those of *Vs*Hb^20^ and sperm whale myoglobin^21^, and even larger ligands with an imidazole moiety bound in a similar pattern to the haem, such as the econazole molecules bound to the structures of FHP^8^ and Yhb^5^, have also been observed. The distal site of *N*-HGbRL is lined by all hydrophobic residues (Phe27, Leu31, Phe42, Leu53, Val56, Val94 and Leu98) except for Tyr28(B10) and Lys52(E10). The A-ring propionate of the haem also hydrogen-bonds to the side chains of Arg80 and Tyr84, and the D-ring propionate to Lys43 N and the Tyr28-bound water W1 (Fig. 3b).

Further optimization subsequently yielded crystals of the closed form, also in the same space group *C*2 but with only a subunit in the asymmetric unit. Intriguingly, the first turn of helix E in the distal site has unravelled, allowing Lys52(E10) to close in to bind to the haem at the sixth coordination site and to a water molecule, W2 (Fig. 3c). Tyr28(B10), meanwhile, has been driven away to hydrogen-bond to both the N of Arg51(E9) and Lys52(E10), as well as to W1. Proposed as a mechanism to regulate ligand affinity, haem hexacoordination has been widely observed in several globin structures including nonsymbiotic plant haemoglobin^9^, fruit fly haemoglobin^22^, *Caenorhabditis elegans* neural globin GLB-6^23^, cytoglobin^24^ and neuroglobin^25^,^26^ that normally involves a distal His(E7) residue, or in truncated haemoglobin^27^ an His(E10) residue. Haem coordination by lysine, however, has thus far been reported only in the structures of cytochrome *c* nitrite reductase (NrfA)^28^ and tetrathionate reductase^29^, but both in the proximal site. Presently the only structures of haem hexacoordination by a proximal histidine and a distal lysine are found in a complex of NrfA and NrfH, a membrane-bound cytochrome *c* quinol dehydrogenase^30^, as well as in the M100K mutant of cytochrome *c*-550 from *Paracoccus versutus*, whose haem-coordinating Met100 was replaced by lysine^31^. The closed form of *N*-HGbRL is hence the first globin structure with Lys–His hexacoordination.

### Distal site reorganization and a large conformational shift upon ligand binding

Both subunits A* of the open and closed structures superpose well at helices A–E (rmsd 1.22 Å) and considerably better at helices F–H (rmsd 0.46 Å), with the distal site undergoing some conformational changes. A ~ 25° bending of helix E results in a large shift of subunits B* between the two forms (Fig. 4a), creating a huge pocket of about 250 Å^3^ in the distal site of the open form that connects to several tunnels (Fig. 4b). In the closed form, meanwhile, Lys48(E6)–Arg51(E9) are untangled and no longer form the first turn of helix E, which tilts towards the haem and binds to it (Fig. 4c). Loop CE is also pulled towards helices B and C, stabilized by new hydrogen bonds between Arg39(C5) from helix C and Phe42–Ile44, between Gly50(E8) from the unravelled turn of helix E and Ile46, and between Arg29(B11) from helix B and Glu47 (Fig. 4d). The side chain of Trp59(E17) has two alternate conformations in the closed form — one with an occupancy of 0.6 facing the solvent and another of 0.4 flipping 180° inwards — but in the open form it flips inwards, appearing to act as a switch between the two forms (Fig. 4e). In the open form, interaction between subunits B* from two adjacent asymmetric units, Arg39(C5) from one asymmetric unit and Glu47 from the other, causes loop CE to shift relative to that of subunit A* (Supplementary Fig. S1).

These changes provide a mechanical insight into how *N*-HGbRL may sense a signal. When a signalling ligand binds the haem in the closed form, it dislodges Lys52(E10) and pushes helix E outwards, inducing part of loop CE to fold into the extra turn of helix E of the open form (Fig. 4c). This may probably expose Trp59(E17) wholly to the solvent and prompt it to flip inwards, further facilitating helices E and F to move away from helix A (Fig. 4a). The side chain of Asp68(E26) breaks its bonding with Arg3 and in turn binds to Arg75(F2), promoting the bending of the fused helices E and F (Fig. 4e). The presence of cooperativity between the two subunits can also be deduced from these structures — the conformational changes induced by ligand binding in subunit A* drives helix F in subunit B* towards helix E, forcing the latter’s fused helices to bend and hence to trigger the reorganization that opens up the closed distal site of subunit B* (Fig. 4a). Once the ligand dissociates, loop CE may tend to assume the closed conformation as it is stabilized by the interactions, absent in the open form, with Arg29(B11), Arg39(C5) and Gly50(E8) that pulls Lys52(E10) towards the haem (Fig. 4d).

### A new globin subgroup

The huge pocket of *N*-HGbRL’s open structure may be able to bind a comparatively large ligand. FHP likewise has a very huge pocket and forms an open and closed state, characterized by rearrangement of loop CE, an extra turn of helix E and a reorientation of the C-terminal NADH domain in the open state^6^,^8^. However, FHP’s reportedly ‘closed’ state is not totally closed — its two closed structures still bind some large azole derivatives — and it is also unclear if FHP’s helix E can move further inwards to bind to the haem when an exogenous ligand is actually absent. On the other hand, HMP has been solved only in a completely closed structure, with its haem totally shielded by Leu57(E11)^11^. The single-domain *Vs*Hb also shields its haem with Leu57(E11) in the closed form, but only slight movements of its helix E and a sideways rotation of Leu57(E11) are already sufficient to open up its distal site to bind a molecule of azide, thiocyanate or imidazole^19^,^20^. Nevertheless, as HMP’s Leu57(E11) is closer to the haem than *Vs*Hb’s Leu57(E11) is, in HMP it may require a larger outward movement of helix E for ligand binding.

*N*-HGbRL and these multi-domain globins probably constitute a subgroup of novel bacterial globins that share distinct features such as an open and a closed form, a huge distal pocket, haem hexacoordination or shielding, and, upon ligand binding, reorganization of loop CE and helix E which may possibly lead to a large movement of a neighbouring domain. A signature of this subgroup may be haem hexacoordination or shielding with a residue at site E10 or E11, instead of E7, such as Lys52(E10) in *N*-HGbRL and Leu57(E11) in HMP. Haem hexacoordination normally involves the invariant His(E7), and only recently has an E11 residue been reported, i.e. His66(E11) in *Geobacter sulfurreducens* globin-coupled sensor (*Gs*GCS)^32^. These globins notably do not possess helix D, whereas globins with haem-coordinating His(E7) usually do. The absence of helix D may not only increase the flexibility in this region, but a shorter sequence may also be more energetically favoured for a concerted conformational change involving loop CE and helix E. Still, at present as only *N*-HGbRL has been solved in both forms, more structures of globins in either form are needed for further studies.

### Architectural differences between *N*-HGbRL and HGbI

**Figure 1:**
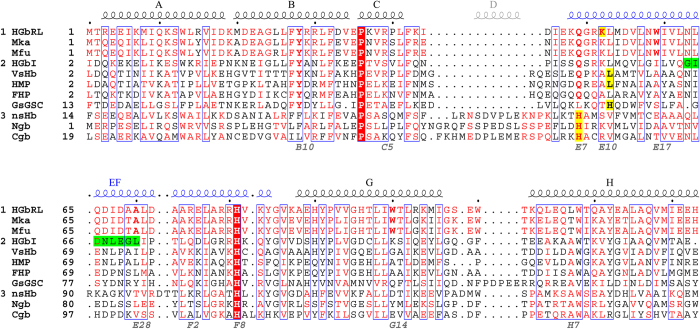
Structural alignments of globins. HGbRL is unique to the genus *Methylacidiphilum* (Group 1), presently found only in two other bacteria, *M. kamchatkense* (Mka) and *M. fumariolicum* (Mfu) (aligned by protein sequences). Its globin domain is closely related to bacterial globins (Group 2) with conserved Tyr(B10) and Gln(E7), as well as lacking helix D that is present in eukaryotic globins (Group 3). Thus far *N*-HGbRL is the only globin containing fused helices E and F (EF) among all the known structures, with the corresponding loop EF of HGbI (green) straightened into part of the elongated helix. Haem hexacoordination in eukaryotic globins invariably involves His(E7) (yellow), as seen in nonsymbiotic plant haemoglobin (nsHb), neuroglobin (Ngb) and cytoglobin (Cgb). In bacterial globins, on the other hand, hexacoordination involves residues at either position E10 or E11, such as Lys52(E10) in *N*-HGbRL, His66(E11) in *Gs*GSC, and Leu57(E11) in both *Vs*Hb and HMP for haem shielding. Following the conventional numbering for the distal Gln/His(E7) and proximal His(F8), the fused helices E and F are numbered E6–E31 followed by F1–F11.

Despite an identity of 38%, the unsuspected large structural differences between *N*-HGbRL and HGbI^3^ have eclipsed their sequence closeness. HGbI contains several buried residues [Gly79(F5), Ser102(G15), Phe111, Ala115(H4), Ala118(H7)] which are replaced by larger residues [Ala78(F5), Thr101(G15), Trp110, Leu114(H4), Leu117(H7)], or solvent-exposed residues [Ile60(E17), Lys101(G14)] by hydrophobic residues [Trp59(E17), Trp100(G14)], in *N*-HGbRL (Fig. 1). This could possibly render an HGbI-like fold unstable for *N*-HGbRL. Nevertheless, helices F–H from both globins superpose considerably well with an rmsd of 0.76 Å (Fig. 4a), and their proximal sites are totally conserved. When comparing their highly conserved Gln(E7)–Leu(E15) regions, however, helix E of *N*-HGbRL’s open form is moved forwards by about a helical turn relative to that of HGbI, leading to a structural ‘frameshift’ of three residues. The O_2_-binding Gln50(E7) of HGbI, for instance, is structurally aligned not to *N*-HGbRL’s corresponding Gln49(E7) but to Lys52(E10), and HGbI’s Val57(E14) is aligned to *N*-HGbRL’s Trp59(E17), which may have a role as a pivot for the shift (Fig. 5a). The shift has lengthened *N*-HGbRL’s loop CE and enlarged its distal site. An interlocking pair of Arg30(B12)–Trp100(G14) has also been observed to shift *N*-HGbRL’s helix B by about half a helical turn (Fig. 5b). The side chains of Trp100(G14) and Thr101(G15) further push helix B outwards, resulting in a similar shift in helix A of a helical turn which is coupled to the shift in helix E.

Other strategic substitutions in *N*-HGbRL, particularly around Asn63(E21)–Ala70(E28) corresponding to HGbI’s loop EF, are also employed for the formation and accommodation of the fused helices E and F. The conversion of HGbI’s Ser51(E8) to Gly50(E8) has not only added flexibility for helix E’s unwinding, but also avoided clashes with helix B. Substituting HGbI’s Gly64 and Gly70 with Asn63(E21) and Ala69(E27), in turn, increases the propensity for helix formation in loop EF, while replacing HGbI’s conserved Pro73 with Asp72(E30) also contributes to helix forming by providing a free backbone N (Fig. 5c). Arg75(F2), a lengthened substitution for HGbI’s Gln76, notably helps straighten loop EF by binding to Asp68(E26) and Asp72(E30) (Fig. 5c). Other residues such as Ile67(E25), Ala70(E28), Ala73(E31) and Ala74(F1) that replace HGbI’s larger Leu68, Leu71, Thr74 and Leu75, meanwhile, are better accommodated in their surroundings. Clashes between the antiparallel pair of helices EF in the dimer are avoided, in particular, by the Ile67(E25)–Ile67(E25) pair in the closed form and the Ala70(E28)–Ala70(E28) pair in the open form (Fig. 5c). Meanwhile, the lengthened loop CE as well as the bulky Arg29(B11) and Arg39(C5), which replace HGbI’s Ala30(B11) and Ser40(C5) and help stabilize loop CE in the closed form, have abolished the mostly hydrophobic dimer interface of HGbI, which is made up of helices B from each subunit (Supplementary Fig. S2).

**Figure 2:**
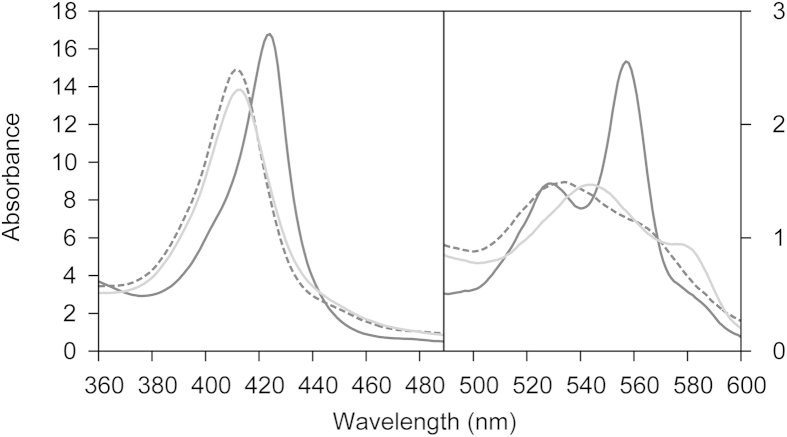
Absorption spectra. Ferric *N*-HGbRL after purification (dashed line) is reduced to the deoxy form (black) in an excess of sodium dithionite, which gradually turns into the oxy form after 20 minutes (grey).

**Figure 3:**
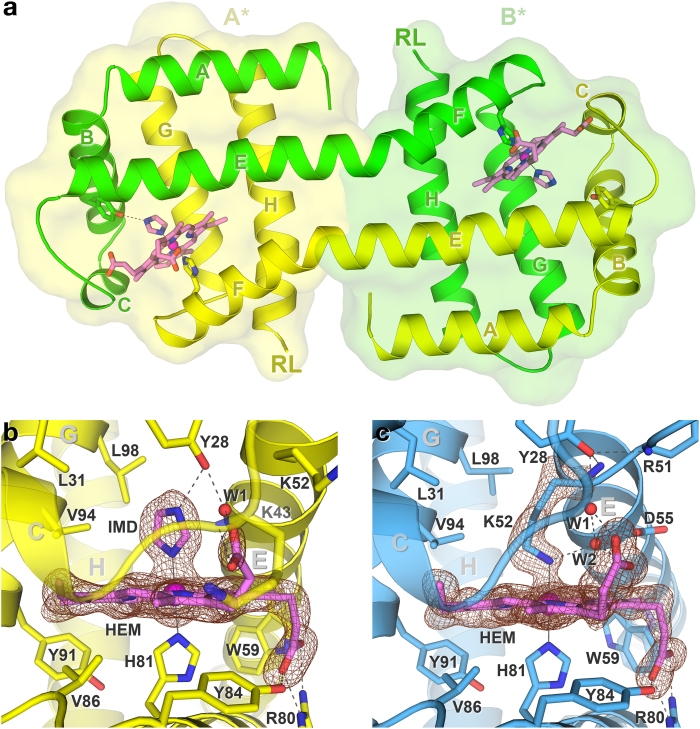
Structures of *N*-HGbRL in the open and closed forms. (**a**) Site-swapped dimer of *N*-HGbRL. Both helices E and F unexpectedly fuse into an elongated helix, resulting in a globin fold, denoted A*, being made up of helices A–E from one subunit (green) and F–H from the other (yellow). While the two C-terminal Roadblock/LC7 (RL) domains are positioned at the opposite ends, they may still lie on the same side to interact with each other. (**b**) Open form of *N*-HGbRL. An imidazole (IMD) molecule binds to the haem (HEM) and the conserved Tyr28(B10). (**c**) Closed form of *N*-HGbRL. Helix E moves inwards and unravels its first helical turn for Lys52(E10) to bind to the haem, forming a unique Lys–His hexacoordinated structure. The *F*_o_ − *F*_c_ omit maps for both structures are contoured at 3 *σ*.

**Figure 4:**
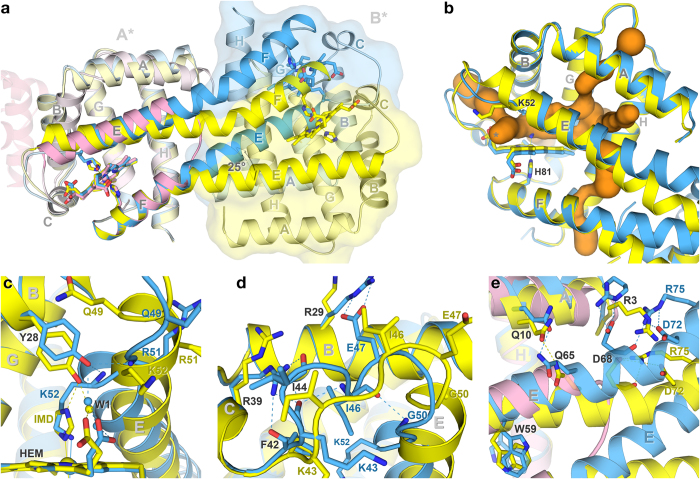
Conformational changes between the open and closed forms. (**a**) Superposition of open (yellow) and closed (blue) forms of subunits A* of *N*-HGbRL with HGbI (pink) at helices F–H. A bending of ~25° of the fused helices E and F leads to a large shift in subunits B* of the two *N*-HGbRL forms. Dimerization of HGbI involves helices B of the first and the second (transparent) subunits. (**b**) Tunnels and distal pocket of *N*-HGbRL. Besides connecting to several tunnels (orange), the pocket in the distal site of the open form is also directly exposed to the solvent through an opening (*). (**c**) Conformational changes in distal site. Helix E moves outwards in the open form to allow Tyr28(B10) to bind to imidazole, whereas it moves towards the haem in the closed form and drives Tyr28(B10) away from the distal site. (**d**) Conformational changes in loop CE. While folding compactly into the distal site in the closed form, loop CE as well as helix E in the open form extend outwards to open up the distal site. (**e**) Conformational changes along the fused helices E and F between the two forms.

**Figure 5:**
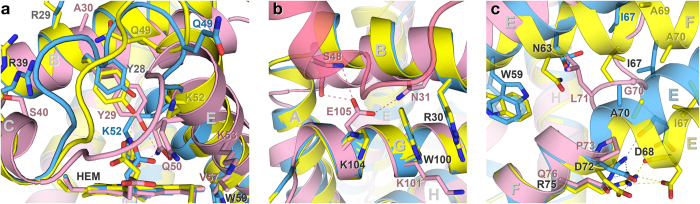
Structural comparison with HGbI. (**a**) Distal site comparison. Despite their highly conserved proximal sites, the distal site of HGbI (pink) differs considerably from those of the open (yellow) and closed (blue) forms of *N*-HGbRL. A forward shift of a helical turn in *N*-HGbRL’s helice E results in the lengthening of loop CE. (**b**) Arg30–Trp100 interlocking pair. The interlocking pair causes *N*-HGbRL’s helix B to move outwards and backwards by half a helical turn, subsequently dragging helix A backwards by about a helical turn which is coupled to the shift in helix E. The similar region in HGbI interacts with the second subunit (transparent). (**c**) Straightening of loop EF. Strategic residue replacements in *N*-HGbRL straighten the region corresponding to HGbI’s loop EF, and reduce clashes between the antiparallel pair of the fused helices EF in the dimer.

HGbI also has a smaller distal pocket which is connected to the solvent through several tunnels^3^, but the distal pocket of the open form of *N*-HGbRL is readily accessible from the solvent through an opening (Fig. 4b). In HGbI’s pocket, Tyr29(B10) is positioned closer to the haem and able to bind an O_2_ molecule, which also binds Gln50(E7). Although spectrally ferrous *N*-HGbRL also binds O_2_ (Fig. 2), it is unclear if its corresponding Tyr28(B10) could directly coordinate O_2_, since this would definitely require a larger outward movement of helix E to make room for Tyr28(B10) to move in. On the other hand, the hexacoordinated spectra of HGbI^3^ are a very perplexing observation, as no histidine is present in the vicinity of the distal site and the only lysine nearby, Lys53(E10), is positioned outside the pocket (Fig. 5a). It would require considerable conformational changes of HGbI’s helices B and E for Tyr29(B10) to make way for Lys53(E10) to move in to bind the haem, and this certainly would also disrupt the dimerization of HGbI. A structure of the hexacoordinated HGbI will no doubt prove interesting to reveal perhaps another new mechanism for haem hexacoordination.

### Evolution of HGbRL from HGbI and HRL

As observed in the reorientation of FHP’s NADH domain^8^, the large conformational movement of *N*-HGbRL between its open and closed forms, by the same token, is also expected to trigger changes in HGbRL’s C-terminal Roadblock/LC7 domain. Roadblock/LC7 proteins form one of the light chains of dynein in eukaryotes, a large multisubunit complex that transports cellular cargo along mircrotubules. In bacteria, they constitute an ancient protein superfamily which may function in NTPase regulation^33^. The Roadblock/LC7 protein from *Myxococcus xanthus*, MglB, is the cognate GTPase-activating protein (GAP) of the GTPase MglA, and they are involved in regulating the dynamic switching of cell polarity in bacterial motility^34^,^35^. The *M. infernorum* genome also encodes a pair of genes similar to the MglB–MglA operon, but whose functions are still unknown — a single-domain Roadblock/LC7 protein (GenBank: ACD84470) 34% identical to the C-terminal domain of HGbRL, and an MglA-like GTPase (GenBank: ACD84469) encoded 183 bp upstream.

HGbRL is unique to the genus *Methylacidiphilum*, and could have evolved from the duplication and subsequent fusion of the genes of HGbI and the Roadblock/LC7 protein. With both domains having their own carbon copies in the genome — hence able to relieve themselves of the same functions — HGbRL was therefore free to have developed a new role of its own. Its globin domain, upon binding a yet to be identified ligand, is likely to mechanistically relay the signal to the Roadblock/LC7 domain through the former’s conformational changes. The downstream pathway might involve cell motility as mediated by the Roadblock/LC7 domain, but *M. infernorum* has been reported as a non-motile rod^1^. Alternatively, since HGbRL is also encoded in the same operon as an S-adenosyl methionine (SAM)-dependent methyltransferase (GenBank: ACD83143), signalling from HGbRL may perhaps lead to the methylation of a target molecule by the methyltransferase. Further characterization of this protein, including identification of the native ligand for the globin domain, will definitely help relate the unusual structural features of HGbRL to its function.

## Methods

### Cloning, protein expression and purification

The gene encoding *N*-HGbRL (residues 1–133; GenBank: ACD83144) was amplified by PCR using primers containing the NdeI and BamHI restriction sites, with a His-tag sequence inserted before the stop codon. The PCR product was cloned into the pET-3a expression vector (Novagen), and the resulting vector was transformed into Rosetta 2(DE3)pLysS (Novagen). The cells were grown in LB medium containing 100 μg/ml ampicillin and 34 μg/ml chloramphenicol. Expression was induced with 50 μM isopropyl-*β*-D-thiogalactopyranoside, supplemented with 100 mg/l FeSO_4_•7H_2_O and 17 mg/l *δ*-aminolevulinic acid. After overnight incubation at 37 °C, the cells were harvested by centrifugation and resuspended in 50 mM Tris-HCl, 200 mM NaCl, pH 8.0, sonicated and centrifuged to remove cell debris. The supernatant was then applied to a Ni^2+^-charged HiTrap IMAC HP column (GE Healthcare), and *N*-HGbRL was eluted with a gradient of up to 1.0 M imidazole. Fractions containing the protein were pooled and further purified by size-exclusion chromatography on a Superdex 200 column (GE Healthcare).

### Absorption spectra

Extensive buffer exchange was carried out to remove traces of imidazole. The absorption spectrum for the met form of *N*-HGbRL was measured in 50 mM Tris-HCl, pH 8.0 at room temperature. The deoxy form was obtained by adding freshly prepared sodium dithionite to a final concentration of 100 mg/ml, and the absorption spectra were measured every ten minutes for an hour.

### Crystallization and data collection

*N*-HGbRL was concentrated to 10–20 mg/ml prior to crystallization. Using the sitting-drop vapour diffusion technique, the imidazole-bound crystals were formed at 20 °C in 3.5 M 1,6-hexanediol, 0.1 M sodium citrate pH 5.6, and cryoprotected with 12% glycerol. Crystals of the hexacoordinated closed form, meanwhile, were formed at 4 °C on a big salt crystal in 50% 2-methyl-2,4-pentanediol, 0.1 M Tris-HCl pH 8.0, 0.2 M (NH_4_)_2_HPO_4_. Diffraction data for both types of crystals, flash-cooled at 100 K, were collected on a Rigaku MicroMax-007 HF X-ray generator (*λ* = 1.5418 Å) equipped with an R-AXIS IV^++^ area detector, and processed with XDS^36^ in the same space group *C*2 but with different cell parameters.

### Structure determination

The HGbI structure^3^ (PDB: 3S1I) was used as an initial model to solve the closed form by molecular replacement with Molrep^37^, which was then used to solve the imidazole-bound form. These structures were subsequently built and refined using Phenix^38^ and Coot^39^, with TLS refinement introduced into the closed form. Data collection and refinement statistics are summarized in Table 1. Structural alignments were performed with the Dali server^40^, manually edited and presented with ESPript^41^. Tunnels and the distal pocket were calculated with CAVER^42^, and figures were generated using PyMOL (http://www.pymol.org).

## Additional Information

**Accession codes:** Atomic coordinates have been deposited with the Protein Data Bank under accession codes 3WFW for the hexacoordinated closed form, and 3WFX for the imidazole-bound open form. 

**How to cite this article**: Teh, A.-H. *et al*. Open and Lys–His Hexacoordinated Closed Structures of a Globin with Swapped Proximal and Distal Sites. *Sci. Rep*. **5**, 11407; doi: 10.1038/srep11407 (2015).

## Supplementary Material

Supplementary Information

## Figures and Tables

**Table 1:** Data collection and refinement statistics.

	Open	Closed
**Data collection**		
Space group	*C*2	*C*2
Cell dimensions		
** ** *a*, *b*, *c* (Å)	98.6, 44.3, 81.9	67.2, 54.4, 44.7
** ** *β* (°)	119.7	106.2
Resolution (Å)	1.94 (1.99–1.94)	1.65 (1.69–1.65)
Completeness (%)	94.3 (99.4)	89.6 (87.3)
Redundancy	2.7 (2.6)	2.3 (2.1)
Mean *I*/*σ*(*I*)	13.9 (3.0)	22.0 (11.0)
*R*_merge_	0.044 (0.307)	0.025 (0.061)
**Refinement**		
No. of reflections	21,703	16,812
*R*_work_	0.183	0.151
*R*_free_	0.234	0.169
R.m.s.d bonds (Å)	0.007	0.009
R.m.s.d angles (°)	1.027	1.201
Mean *B*-factors (Å^2^)		
** ** Protein	25.8	18.7
** ** Ligands	20.4	16.6
** ** Water	32.1	30.6
Ramachandran plot		
** ** Favoured (%)	98.6	98.6
** ** Allowed (%)	1.4	1.4
Values for the outer shell are given in parentheses.		

## References

[b1] Dunfield P. F. . Methane oxidation by an extremely acidophilic bacterium of the phylum Verrucomicrobia. Nature 450, 879–882 (2007). 18004300 10.1038/nature06411

[b2] Hou S. . Complete genome sequence of the extremely acidophilic methanotroph isolate V4, *Methylacidiphilum infernorum*, a representative of the bacterial phylum *Verrucomicrobia*. Biol. Direct 3, 26 (2008). 18593465 10.1186/1745-6150-3-26 PMC2474590

[b3] Teh A. H. . Hell’s Gate globin I: an acid and thermostable bacterial hemoglobin resembling mammalian neuroglobin. FEBS Lett. 585, 3250–3258 (2011). 21925500 10.1016/j.febslet.2011.09.002

[b4] Jamil F. . Crystal structure of truncated haemoglobin from an extremely thermophilic and acidophilic bacterium. J. Biochem. 156, 97–106 (2014). 24733432 10.1093/jb/mvu023

[b5] El Hammi E. . Active site analysis of yeast flavohemoglobin based on its structure with a small ligand or econazole. FEBS J 279, 4565–4575 (2012). 23095020 10.1111/febs.12043

[b6] Ermler U., Siddiqui R. A., Cramm R. & Friedrich B. Crystal structure of the flavohemoglobin from *Alcaligenes eutrophus* at 1.75 Å resolution. EMBO J. 14, 6067–6077 (1995). 8557026 10.1002/j.1460-2075.1995.tb00297.x PMC394731

[b7] Ollesch G., Kaunzinger A., Juchelka D., Schubert-Zsilavecz M. & Ermler U. Phospholipid bound to the flavohemoprotein from *Alcaligenes eutrophus*. Eur. J. Biochem. 262, 396–405 (1999). 10336624 10.1046/j.1432-1327.1999.00381.x

[b8] El Hammi E. . Structure of *Ralstonia eutropha* flavohemoglobin in complex with three antibiotic azole compounds. Biochemistry 50, 1255–1264 (2011). 21210640 10.1021/bi101650q

[b9] Hargrove M. S. . Crystal structure of a nonsymbiotic plant hemoglobin. Structure 8, 1005–1014 (2000). 10986467 10.1016/s0969-2126(00)00194-5

[b10] Vallone B., Nienhaus K., Matthes A., Brunori M. & Nienhaus G. U. The structure of carbonmonoxy neuroglobin reveals a heme-sliding mechanism for control of ligand affinity. Proc. Natl. Acad. Sci. U. S. A. 101, 17351–17356 (2004). 15548613 10.1073/pnas.0407633101 PMC536024

[b11] Ilari A., Bonamore A., Farina A., Johnson K. A. & Boffi A. The X-ray structure of ferric *Escherichia coli* flavohemoglobin reveals an unexpected geometry of the distal heme pocket. J. Biol. Chem. 277, 23725–23732 (2002). 11964402 10.1074/jbc.M202228200

[b12] Poole R. K. & Hughes M. N. New functions for the ancient globin family: bacterial responses to nitric oxide and nitrosative stress. Mol. Microbiol. 36, 775–783 (2000). 10844666 10.1046/j.1365-2958.2000.01889.x

[b13] Hou S. . Myoglobin-like aerotaxis transducers in Archaea and Bacteria. Nature 403, 540–544 (2000). 10676961 10.1038/35000570

[b14] Marles-Wright J. . Molecular architecture of the “stressosome,” a signal integration and transduction hub. Science 322, 92–96 (2008). 18832644 10.1126/science.1159572

[b15] Murray J. W., Delumeau O. & Lewis R. J. Structure of a nonheme globin in environmental stress signaling. Proc. Natl. Acad. Sci. U. S. A. 102, 17320–17325 (2005). 16301540 10.1073/pnas.0506599102 PMC1297668

[b16] Saw J. . Complete genome sequencing and analysis of *Saprospira grandis* str. Lewin, a predatory marine bacterium. Stand. Genomic Sci. 6, 84–93 (2012). 22675601 10.4056/sigs.2445005 PMC3368406

[b17] Islam T., Jensen S., Reigstad L. J., Larsen O. & Birkeland N. K. Methane oxidation at 55°C and pH 2 by a thermoacidophilic bacterium belonging to the *Verrucomicrobia* phylum. Proc. Natl. Acad. Sci. U. S. A. 105, 300–304 (2008). 18172218 10.1073/pnas.0704162105 PMC2224206

[b18] Khadem A. F. . Draft genome sequence of the volcano-inhabiting thermoacidophilic methanotroph *Methylacidiphilum fumariolicum* strain SolV. J. Bacteriol. 194, 3729–3730 (2012). 22740660 10.1128/JB.00501-12 PMC3393509

[b19] Tarricone C., Galizzi A., Coda A., Ascenzi P. & Bolognesi M. Unusual structure of the oxygen-binding site in the dimeric bacterial hemoglobin from *Vitreoscilla* sp. Structure 5, 497–507 (1997). 9115439 10.1016/s0969-2126(97)00206-2

[b20] Bolognesi M. . Anticooperative ligand binding properties of recombinant ferric *Vitreoscilla* homodimeric hemoglobin: a thermodynamic, kinetic and X-ray crystallographic study. J. Mol. Biol. 291, 637–650 (1999). 10448042 10.1006/jmbi.1999.2975

[b21] Lionetti C., Guanziroli M. G., Frigerio F., Ascenzi P. & Bolognesi M. X-ray crystal structure of the ferric sperm whale myoglobin: imidazole complex at 2.0 Å resolution. J. Mol. Biol. 217, 409–412 (1991). 1994031 10.1016/0022-2836(91)90744-q

[b22] de Sanctis D. . Bishistidyl heme hexacoordination, a key structural property in *Drosophila melanogaster* hemoglobin. J. Biol. Chem. 280, 27222–27229 (2005). 15917230 10.1074/jbc.M503814200

[b23] Yoon J. . Structure and properties of a bis-histidyl ligated globin from *Caenorhabditis elegans*. Biochemistry 49, 5662–5670 (2010). 20518498 10.1021/bi100710a PMC2903878

[b24] de Sanctis D. . Crystal structure of cytoglobin: the fourth globin type discovered in man displays heme hexa-coordination. J. Mol. Biol. 336, 917–927 (2004). 15095869 10.1016/j.jmb.2003.12.063

[b25] Pesce A. . Human brain neuroglobin structure reveals a distinct mode of controlling oxygen affinity. Structure 11, 1087–1095 (2003). 12962627 10.1016/s0969-2126(03)00166-7

[b26] Vallone B., Nienhaus K., Brunori M. & Nienhaus G. U. The structure of murine neuroglobin: Novel pathways for ligand migration and binding. Proteins 56, 85–92 (2004). 15162488 10.1002/prot.20113

[b27] Hoy J. A., Kundu S., Trent J. T. 3rd, Ramaswamy S. & Hargrove M. S. The crystal structure of *Synechocystis* hemoglobin with a covalent heme linkage. J. Biol. Chem. 279, 16535–16542 (2004). 14736872 10.1074/jbc.M313707200

[b28] Einsle O. . Structure of cytochrome *c* nitrite reductase. Nature 400, 476–480 (1999). 10440380 10.1038/22802

[b29] Mowat C. G. . Octaheme tetrathionate reductase is a respiratory enzyme with novel heme ligation. Nat. Struct. Mol. Biol. 11, 1023–1024 (2004). 15361860 10.1038/nsmb827

[b30] Rodrigues M. L., Oliveira T. F., Pereira I. A. & Archer M. X-ray structure of the membrane-bound cytochrome *c* quinol dehydrogenase NrfH reveals novel haem coordination. EMBO J. 25, 5951–5960 (2006). 17139260 10.1038/sj.emboj.7601439 PMC1698886

[b31] Worrall J. A., van Roon A. M., Ubbink M. & Canters G. W. The effect of replacing the axial methionine ligand with a lysine residue in cytochrome *c*-550 from *Paracoccus versutus* assessed by X-ray crystallography and unfolding. FEBS J. 272, 2441–2455 (2005). 15885094 10.1111/j.1742-4658.2005.04664.x

[b32] Pesce A. . HisE11 and HisF8 provide bis-histidyl heme hexa-coordination in the globin domain of *Geobacter sulfurreducens* globin-coupled sensor. J. Mol. Biol. 386, 246–260 (2009). 19109973 10.1016/j.jmb.2008.12.023

[b33] Koonin E. V. & Aravind L. Dynein light chains of the Roadblock/LC7 group belong to an ancient protein superfamily implicated in NTPase regulation. Curr. Biol. 10 R774–776 (2000). 11084347 10.1016/s0960-9822(00)00774-0

[b34] Leonardy S. . Regulation of dynamic polarity switching in bacteria by a Ras-like G-protein and its cognate GAP. EMBO J. 29, 2276–2289 (2010). 20543819 10.1038/emboj.2010.114 PMC2910265

[b35] Zhang Y., Franco M., Ducret A. & Mignot T. A bacterial Ras-like small GTP-binding protein and its cognate GAP establish a dynamic spatial polarity axis to control directed motility. PLoS Biol. 8, e1000430 (2010). 20652021 10.1371/journal.pbio.1000430 PMC2907295

[b36] Kabsch W. XDS. Acta Crystallogr. D Biol. Crystallogr. 66, 125–132 (2010). 20124692 10.1107/S0907444909047337 PMC2815665

[b37] Vagin A. & Teplyakov A. Molecular replacement with MOLREP. Acta Crystallogr. D Biol. Crystallogr. 66, 22–25 (2010). 20057045 10.1107/S0907444909042589

[b38] Adams P. D. . PHENIX: a comprehensive Python-based system for macromolecular structure solution. Acta Crystallogr. D Biol. Crystallogr. 66, 213–221 (2010). 20124702 10.1107/S0907444909052925 PMC2815670

[b39] Emsley P., Lohkamp B., Scott W. G. & Cowtan K. Features and development of Coot. Acta Crystallogr. D Biol. Crystallogr. 66, 486–501 (2010). 20383002 10.1107/S0907444910007493 PMC2852313

[b40] Holm L. & Rosenstrom P. Dali server: conservation mapping in 3D. Nucleic Acids Res. 38, W545–549 (2010). 20457744 10.1093/nar/gkq366 PMC2896194

[b41] Gouet P., Courcelle E., Stuart D. I. & Metoz F. ESPript: analysis of multiple sequence alignments in PostScript. Bioinformatics 15, 305–308 (1999). 10320398 10.1093/bioinformatics/15.4.305

[b42] Chovancova E. . CAVER 3.0: a tool for the analysis of transport pathways in dynamic protein structures. PLoS Comput. Biol. 8, e1002708 (2012). 23093919 10.1371/journal.pcbi.1002708 PMC3475669

