# Supplementary material for: Open and Lys–His Hexacoordinated Closed Structures of a Globin with Swapped Proximal and Distal Sites
Source: Sci Rep. 2015 Jun 22;5:11407. doi: 10.1038/srep11407 (PMC4476040; doi:10.1038/srep11407)
Supplement: Supplementary Information [file srep11407-s1.pdf]

Supplementary information

Open and His–Lys Hexacoordinated Closed Structures of a Globin with Swapped Proximal and Distal Sites

Aik-Hong Teh[1], Jennifer A. Saito[2], Nazalan Najimudin[3] & Maqsudul Alam[2,4]

[1]Centre for Chemical Biology, Universiti Sains Malaysia, 10 Persiaran Bukit Jambul, 11900 Bayan Lepas, Penang, Malaysia, [2]Department of Microbiology, University of Hawaii, 2538 McCarthy Mall, Honolulu, HI 96822, United States, [3]School of Biological Sciences, Universiti Sains Malaysia, 11800 Penang, Malaysia, [4]Advanced Studies in Genomics, Proteomics and Bioinformatics, University of Hawaii, 2565 McCarthy Mall, Honolulu, HI 96822, United States.

Correspondence and requests for materials should be addressed to A.H.T. (aikhong@usm.my)

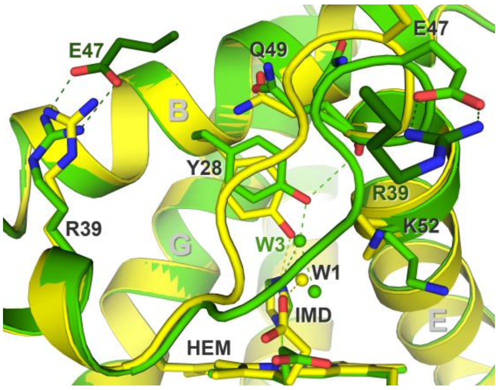

Supplementary Figure S1 │ Conformational changes in subunit B* of the open form. Because of interactions with Arg39(C5) and Glu47 from a neighbouring asymmetric unit (dark green), absent in subunit A* (yellow), a different conformation of loop CE is observed in subunit B* (green) of the open form. A slight movement of helix E is also observed, allowing Tyr28(B10) in the distal site to move to an upward position similar to that of the closed form, and W3 to move in.

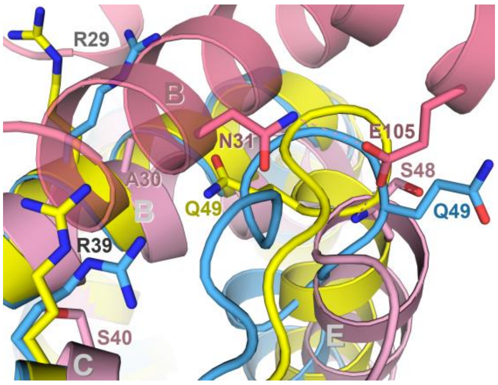

Supplementary Figure S2 │ Absence of HGbI dimerization in *N*-HGbRL. In both the open (yellow) and closed (blue) forms of *N*-HGbRL, the lengthened loop CE and several residue replacements, such as the bulky Arg29(B11) and Arg39(C5), have disrupted the dimeric interface present in HGbI (pink; second subunit in dark pink).
